# Female genital mutilation: prevalence, associated factors and health consequences among reproductive age group women in Keffa Zone, Southwest, Ethiopia

**DOI:** 10.1186/s12978-022-01364-3

**Published:** 2022-03-05

**Authors:** Tesfaye Abera Gudeta, Tilahun Mekonnen Regassa, Lalisa Chewaka Gamtessa

**Affiliations:** 1 Department of Nursing, Institute of Health Sciences, Wollega University, Nekemte, Ethiopia; 2 Department of Nursing, College of Health Sciences, Mizan-Tepi University, Mizan, Ethiopia

**Keywords:** Female genital mutilation, Women reproductive age group, Keffa zone, Ethiopia

## Abstract

**Background:**

Female genital mutilation is procedure involving partial or total removal of the external female genitalia for cultural or non-therapeutic reasons. Despite of global concerns, awareness, and campaigns, the prevalence of female genital mutilation remains high in many countries.

**Objective:**

To assess female genital mutilation: prevalence, associated factors, and health consequences among reproductive age group women in Keffa Zone, Southwest, Ethiopia.

**Methods:**

A community based cross-sectional study design was employed from March 01 to April 30, 2019. We used a multi stage sampling. Finally, using simple random sampling technique, we selected four Woredas. Consequently, after clustering kebeles, all mothers with daughter/s younger than 15 years and live in clustered kebeles were interviewed. Data were entered into Epi data and exported to SPSS version 23.0. Variables with P-value of less than 0.25 in binary logistic regression analyses were entered into the multivariable logistic regression analysis. Odds ratio with 95% confidence interval was used to determine associations between dependent and independent variables. P value less than 0.05 was considered statistically significant.

**Results:**

Almost all, 729 (97.2%) of mothers expressed that they heard about female circumcision. However, less than one-fourth, 159 (21.2%) of mothers were circumcised. Among those 159 women ever circumcised, 52 (32.7%) reported that they experienced the complication of female genital mutilation.The prevalence of female genital mutilation of daughters’ younger than 15 years was 12 (1.6%). Rural residence [AOR 6.74, 95% CI (2.70–16.85)], being Muslim and Protestant follower by their religion [AOR 0.19, 95% CI (0.07–0.53] and [AOR 0.54, 95% CI (0.30–0.98)] respectively and occupational status of the husband; Merchant [AOR 7.29; 95% CI (3.66–14.51)], Daily laborer[AOR 2.70, 95% CI (1.14–6.40)] and others (drivers and students) [AOR 6.70, 95% CI (1.55–28.95] were statistically significantly associated with female genital mutilation.

**Conclusion:**

In this study, prevalence of female genital mutilation practice among daughters and women of reproductive group was low as compared to a national data. However, that much magnitude still seeks attention as Ethiopia planned to end the practice. Religion, place of residence, and occupational status of the husband were statistically significantly associated with female genital mutilation. Therefore, any strategy to end up the practice better considers dimension of all these variables.

## Plain English Summary

Female Genital Mutilation is a procedure involving partial or total removal of the external female genitalia for cultural or non-therapeutic reasons. The female genital mutilation is a practice associated with a variety of socio-cultural meanings and often considered as necessary for social acceptability. Despite global concerns, awareness and campaigns, the prevalence of female genital mutilation remains high in many countries. In spite of the prohibition practice, ending female genital mutilation is still a problem as the practice is conducted secretly and quietly.

It is important to know the prevalence and associated factors of female genital mutilation in the region to work on the problem. In this study, we have interviewed reproductive age group women in the community to explore the practice and associated factors of female genital mutilation among themselves and their daughters.

This study indicates that there is low prevalence of female genital mutilation in the region as compared to other region and national prevalence. Even though the practice is low in the region, it needs an attention to ending up the practice. This study findings suggests that deep study is needed to explore the connection between religion and female genital mutilations

## Introduction

World Health Organization defines female genital mutilation (FGM) as all procedures involving partial or total removal of the external female genitalia for cultural or non-therapeutic reasons. FGM is classified into four types; type I is partial or total removal of the clitoris and/or the prepuce (clitoridectomy), type II is a partial or total removal of the clitoris and labia minora, with or without excision of the labia majora (excision), type III involves narrowing of the vaginal orifice by creation of a covering seal through cutting of labia minora and/or the labia majora and type IV is all other harmful procedures to the female genitalia for nonmedical purposes [1, 2].

Female genital mutilation is generally performed by a traditional practitioner. This traditional harmful practice was associated with a variety of socio-cultural meanings and it is considered necessary for social acceptability. Nevertheless, the practice is invasive and associated with severe pain and both immediate and long term health risks, and violates women’s fundamental human rights [3].

In spite of global concerns, awareness, and campaigns, prevalence of FGM remains high in many countries. Though exact number remained unknown, the recent estimates by the United Nations Children’s Fund (UNICEF) suggested that at least 200 million girls and women in 30 countries were subjected to the practice [4]. In more than half of the countries, most girls underwent FGM before age 5 year, while the rest conducted it between the age 5 and 14 years [5]. In the same way**,** more than three million girls Africa were estimated to practice FGM, which is equivalent to one FGM practice every 10 s [6].

According to Ethiopian demographic health survey, prevalence of FGM has declined over the last 16 years. Despite of the reduction, it remains a serious concern in Ethiopia affecting 23.8 million women and girls. Even though FGM is practiced everywhere in Ethiopia, the highest prevalence was in Afar (91.6%),in Somali region (97.3%) and in Dire Dawa (92.3%). In contrary, the lowest prevalence was identified in Gambela and Tigray [7–9].

Ethiopia is dedicated to eliminate the practice of FGM by strategic and programmatic measures. One instant measure was to by enactment of a ban against the FGM practice and penalizes the act in the national criminal code revised in 2005. However, there is still a challenge of end the practice as community obscure the practice [10]. The current proportion of FGM practice is very important to develop strategic measures. In contrary, there was no study conducted in the study area (Keffa Zone). Therefore, this study was aimed to assess the current prevalence of FGM practice and factors associated with FGM. Likewise, the finding of this study can be a baseline data for Zonal Health Office to develop strategy toward ending of the practice. Furthermore, the finding of this study can be an input for systematic review to challenge the policy makers.

## Materials and methods

### Study area and study period

The study was conducted from March 01 to April 30, 2019 in Keffa Zone. Keffa Zone is found in the Southern Nations, Nationalities, and Peoples' Region (SNNPR), Ethiopian. Keffa Zone has 11 Woreda and bordered on the South by Debub Omo, on the Southwest by Bench Maji, on the West by Sheka, on the North by the Oromia Region, and on the East by Konta. Gojeb River runs along part of the Northern border of this zone. The administrative Center of Keffa is Bonga town which is located Keffa Zone on 464 km from Addis Ababa, a capital city of Ethiopia, [11]. According to report in 2019 from the health department, the Keffa Zone has estimated 272,491 women reproductive age group. To give more emphasis about the study area, the map of Keffa Zone can be accessed at https://www.google.com/maps/place/Keficho+Shekicho/@7.2439271,35.6979312,10z/data=!4m5!3m4!1s0x17aeb14ede2be253:0xb850aab99bf69021!8m2!3d7.3360745!4d35.7406882?hl=am

### Study design and populations

Community based cross-sectional study design was employed. All women of reproductive age group (15–49) who were living in keffa Zone were considered as a source population. All women in reproductive age group with daughter/s younger than 15 years in the selected woredas were considered as study population.

### Sample size determination and sampling technique

Sample size was determined using single population proportion based on the following assumptions (1.96)^2^ at 95% confidence level for two side, expected prevalence 65% of FGM used to calculate the sample size [9] and margin of error 5%. Then the calculated sample was 350, after multiplying by design effect of “2”, the sample size became 2*350. By considering 10% non-response rate, the final sample size was 770. Multi stage sampling was used and four woredas namely Gimbo, Wacha, Tallo and Shishinede were selected by using simple random sampling technique. After selecting the woredas, kebeles were clustered. Then all mothers with daughter/s younger than 15 years who were living in clustered kebeles were interviewed.

### Inclusion and exclusion criteria

All women in reproductive in reproductive age group with daughter/s younger than 15 and who lived in the kebeles for at least 6 months were included. Women in reproductive age group who were critically ill or mentally incapable to provide information were excluded from the study.

### Operational definition

#### Female genital mutilation

Defined as all procedures involving partial or total removal of the external female genital or other injury to the female genital organs whether for cultural or other non-therapeutic reasons [1].

#### Attitude

It was classified as positive attitude and negative attitude based on mean score. Those women who scored above mean value were considered as having positive attitude for the continuation of this practice. Those women who scored below a mean value were considered as having negative attitude**.**

#### Knowledge

A woman was considered as knowledgeable on health risks of FGM, if the score was greater than mean score and not knowledgeable if scored less than mean score. This was applied for both immediate and chronic health complications of FGM and measured by 8 items and 7 items respectively.

### Data collection technique and tools

The data collection tool was developed from 2016 EDHS and by reviewing different literatures [9, 12–18]. The face validity test was checked by respective experts. Questions were grouped and arranged according to the particular objectives that they can address. Ten data collectors who were female teachers from School in study area were purposefully selected and recruited. We also recruited four supervisors who had BSc in in field of health Sciences. Then trained data collectors applied face to face and interviewed all selected mothers at their home setting.

### Quality control measures

The quality of the data was assured by using validated and pre-tested questionnaires. Prior to the actual data collection, pre-testing was conducted on 5% of the total study eligible subjects and with similar characteristics in non-selected woredas. Likewise, necessary amendments were made to data collection tool. On the top of that, data collectors were trained intensively for two days on data collection tool. Moreover, the data collectors worked under close supervision to ensure quality of collected data. Finally, collected data were carefully entered and cleaned before the running analysis.

### Data processing and analysis

EPI data version 3.1 and SPSS software version 21.0 was used for data entry and analysis. After organizing and cleaning data, frequencies, and percentages were calculated for variables of interests.

Variables with P-value less than 0.25 in binary logistic regression analysis were entered into the multivariable logistic regression analysis. Odds ratio with 95% confidence interval was used to examine associations between dependent and independent variables. P value less than 0.05 was considered as statistically significant.

### Ethical considerations

After obtaining Ethical clearance from Mizan Tepi University, and permission letter was written for us from respective authorities. Verbal consent of each respondent was obtained before the collecting data. The verbal consent ensured their voluntarily participation and right to take part or terminate at any time they decide. In advance, confidentiality and anonymity of all respondents’ responses were all respected.

## Result

### Socio-demographic characteristics

In this study, 770 questionnaires were distributed and 750 returned making it 97.4% response rate. Most of the respondents, 158 (21.1%) participated in this study were between 25 and 39 years of age, were Orthodox 488 (65.1) in religion and from urban residents, 665 (88.7%). Most mothers, 662 (88.3%) were housewives while around two-thirds, 486 (64.8%) of the fathers were farmers in occupation. Concerning education level, only few of the respondents completed above a secondary cycle (Table 1).

**Table 1:** Socio-demographic characteristics study participants in keffa Zone, Southwest, Ethiopia, 2020 (N = 750)

Variable	Category	Frequency	Percent
Age	15–19	14	1.9
	20–24	48	6.4
	25–29	158	21.1
	30–34	202	26.9
	35–39	179	23.9
	40–44	99	13.2
	45–49	50	6.7
Marital status	Single	28	3.7
	Married	658	87.7
	Widowed	44	5.9
	Divorced	20	2.7
Residence	Urban	665	88.7
	Rural	85	11.3
Religion	Orthodox	488	65.1
	Muslim	78	10.4
	Protestant	174	23.2
	Others*	10	1.3
Educational status of mothers	Illiterate	268	35.7
	Primary cycle	335	44.7
	Secondary	67	8.9
	Diploma and above	80	10.7
Occupation of mother	House wife	662	88.3
	Laborer	33	4.4
	Merchant	23	3.1
	Government employ	31	4.1
	NGO	1	0.1
Educational status of husbands	Illiterate	268	35.7
	Primary cycle	335	44.7
	Secondary	67	8.9
	Diploma and above	80	10.7
Occupation of husbands	Farmer	486	64.8
	Merchant	111	14.8
	Laborer	51	6.8
	Government employ	87	11.6
	NGO	3	0.4
	Others**	12	1.6

*Catholic

**Driver, students

### Prevalence of mother’s FGM

Almost all, 729 (97.2%) mothers in the current study expressed that they heard about female circumcision. However, less than one-fourth, 159 (21.2%) of mothers were circumcised and almost all of them were circumcised by traditional personnel. Around half of mothers, 72 (45.3%) didn’t remember the time of their circumcision. However, few of them were quite sure that their circumcision was lately. For the majority mothers, 132 (83%) flesh was removed but none of them had their genital sewn (Table 2).

**Table 2:** Variable related to Women’s female genital mutilation participated in study, KeffaZone SWE, 2019

Variable	Category	Frequency	Percent
Ever heard circumcision	Yes	729	97.2
	No	21	2.8
Ever circumcised	Yes	159	21.2
	No	591	78.8
Type of circumcision	Flesh removed	132	83
	Genital nicked	97	61
Age of mother at circumcision	I don't know	72	45.3
	5–9	49	30.8
	10–14	29	18.2
	≥ 15	9	5.7
Person performed circumcision	Traditional circumciser	157	98.7
	Health professionals	2	1.3

### Prevalence of daughters’ genital mutilation

The prevalence of FGM in daughters’ younger than 15 years was 12 (1.6%). All the circumcisions were performed by traditional practitioner and mostly by removing flesh 10, (83.3%). The majority, 10 (83.3%) of the circumcision was conducted within the first 10 years of life. It was important to note that among circumcised daughters, almost all, 11 (91.6) of them had their mothers circumcised too. The majority, 674 (89.9%) of women expressed that the circumcision has no benefit. But the rest believed that circumcision was useful for hygiene, for social acceptance, and religious approval. The main reason of not undergoing circumcision was due to health impact, 595 (83.6%) and legal prohibition 109 (15.3%) (Table 3).

**Table 3:** Variable related to daughters’ female genital mutilation, Keffa Zone Southwest Ethiopia 2019

Variable	Category	Frequency	Percent
Age less than 15 years daughters circumcised	Yes	12	1.6
	No	738	98.4
Type of circumcision	Flesh removed	10	83.3
	Genital nicked	2	16.7
Daughters’ age at circumcision	< 10 years	10	83.3
	≥ 10 years	2	16.7
Who performed	Traditional circumciser	12	100
Why not circumcised	Her age is not reached	4	0.6
	Has health impact	595	83.6
	Not permitted by law	109	15.3
	Other^¥^	4	0.6
Intention to circumcise in future	No	702	98.6
	I don't know	10	1.4
Benefit girls get from circumcision	Cleanliness	6	0.8
	Social acceptance	42	5.6
	Better marriage prospect	5	0.7
	More sexual pleasure for the man	2	0.3
	Religious approval	17	2.3
	No benefit	674	89.9
	Other**	4	0.5

^¥^ My husband refused, my neighbors protect me

**Maintain virginity, Cultural value

### Health consequences of FGM for circumcised women and daughter

Among 159 women ever circumcised, 52 (32.7%) reported that they had experienced the complication of FGM. Among 52 women who had experienced health consequences of the FGM, excessive bleeding, 46 (88.5%), urine retention, 30 (57.7%), infection, 23 (44.2%), genital swelling, 21 (40.4%), prolonged labour, 23 (44.2), and excessive bleeding during child birth 20 (38.5). Among 12 circumcised daughters, 4 (33.3%) of them developed the health complications of FGM.

### Knowledge of women on FGM

Among study participants, 637 (84.9%) reported that they had information about the health consequences of FGM. Among these women, 469 (62.5%) had good knowledge about immediate health consequences of FGM. Regarding the immediate health consequences of FGM, 529 (83%) women knew about sever pain, 463 (72.7%) women knew about excessive bleeding and 307 (48.2%) women knew about genital tissue swelling.

Concerning the chronic health consequences of FGM, 456 (60.8%) of the participants had good knowledge. Among the chronic health consequences of FGM, 467 (73.3%) of the participants reported that they knew about difficulty of labour and child birth, 332 (52.1%) of the participants knew about chronic reproductive tract information and 327 (51.3%) of them knew difficulty of sexual intercourse (Table 4).

**Table 4:** Knowledge about FGM among women of reproductive age group Keffa Zone, Southwest, Ethiopia, 2019

Variables	Category	Frequency	Percent
Having information about health consequence FGM?	Yes	637	84.9
	No	113	15.1
1. Knowledge about immediate health consequences of FGM			
Severe pain	Yes	529	83.0
	No	108	17.0
Excessive bleeding	Yes	463	72.7
	No	174	27.3
Genital tissue swelling	Yes	307	48.2
	No	330	51.8
Infection and HIV	Yes	339	53.2
	No	298	46.8
Urine retention	Yes	203	31.9
	No	434	68.1
Impaired wound healing	Yes	256	40.2
	No	381	59.8
Psychological consequence	Yes	256	40.2
	No	381	59.8
Death	Yes	133	20.9
	No	504	79.1
Total knowledge of immediate health consequences	Good Knowledge	469	62.5
	Poor knowledge	168	22.4
	have no information	113	15.1
2. Knowledge about chronic health consequences of FGM			
Chronic reproductive tract infection	Yes	332	52.1
	No	305	47.9
Pain full urination	Yes	271	42.5
	No	366	57.5
Menstrual problem	Yes	235	36.9
	No	402	63.1
Difficulty of sexual intercourse	Yes	327	51.3
	No	310	48.7
Difficulty of labour and child birth	Yes	467	73.3
	No	170	26.7
Obstetric fistula	Yes	274	43.0
	No	363	57.0
Psychological problem	Yes	113	15.1
	No	232	30.9
Total knowledge of chronic health consequences	Good Knowledge	456	60.8
	Poor knowledge	181	24.1
	have no information	113	15.1

### Attitude of women towards FGM

The mean score of attitude of women was 25.3 with the minimum and maximum score being 14 and 26 respectively. Based on mean score, more than half, 460 (61.3%) of the participants had negative attitude meaning that they don’t favor the FGM practice and 290 (38.7%) had good attitude towards FGM practice.

### Factors associated with FGM

Using multivariable logistic regression, contributing factors of FGM practice were identified. These predictors were religion, residence and occupational status of the husband.

Women from rural residence were seven times more likely exercised FGM as compared to women in urban residence [AOR 6.74, 95% CI (2.70–16.85)]. Women who were Muslim and Protestant followers were less likely practiced FGM by 81% as compared to their counterparts[AOR 0.19,95% CI (0.07–0.53] and [AOR 0.54, 95% CI (0.30–0.98)] respectively.

Occupational status of the husband was statistically significantly associated with FGM. Being merchant was seven times [AOR 7.29; 95% CI (3.66–14.51)], being daily laborer was three times [AOR 2.70, 95% CI (1.14–6.40)] and being (drivers and students) was seven times [AOR 6.70, 95% CI (1.55–28.95] more likely practiced female genital mutilation as compared to farmer (Table 5).

**Table 5:** Multivariable logistic regression analysis of FGM and effects on women’s health among reproductive age group women in Keffa zone, Southwest, Ethiopia, 2019

Variables	Category	COR (95% CI)	AOR (95% CI)
Marital status	Single	1	1
	Married	0.32 (0.15–0.70)	0.90 (0.22–3.77)
	Widowed	2.23 (0.85–5.88)	0.78 (0.10–1.87)
	Divorced	1.55 (0.485–4.93)	3.34 (0.89–12.60)
Residence	Rural	2.46 (1.20–5.03)	6.74 (2.70–16.85)*
	Urban	1	1
Religion	Orthodox	1	1
	Muslim	0.24 (0.07–0.85)	0.19 (0.07–0.53)*
	Protestant	0.05 (0.01–0.22)	0.54 (0.30–0.98)*
	Other©	0.09 (0.02–0.33)	2.49 (0.59–10.54)
Educational status of mother	Illiterate	0.39 (0.18–0.83)	1.03 (0.31–3.38)
	Primary (1–8)	0.18 (0.08–0.41)	0.39 (0.13–1.22)
	Secondary (9–12)	0.91 (0.39–2.16)	0.76 (0.27–2.17)
	Diploma and above	1	1
Educational status of the husband	Illiterate	0.41 (0.24–0.71)	0.20 (0.02–1.67)
	Primary (1–8)	0.30 (0.17–0.52)	0.18 (0.02–1.42)
	Secondary (9–12)	0.99 ( 0.51–1.94)	0.53 (0.07–3.84)
	Diploma and above	1	1
Occupational status of the husband	Farmer	1	1
	Merchant	2.76 (1.73–4.41)	7.29 (3.66–14.51)*
	Laborer	1.77 (0.88–3.54)	2.70 (1.14–6.40)*
	Govn’t employee	3.35 (2.02–5.53)	0.92 (0.12–7.21)
	Other**	8.05 (2.49–26.06)	6.70 (1.55–28.95)*
Ever heard circumcision	Yes	1	1
	No	5.53 (0.74–41.56)	0.15 (0.02–1.22)
Attitude towards FGM	Positive attitude	1.56 (1.10–2.22)	1.40 (0.89–2.20)
	Negative attitude	1	1
Knowledge of women on immediate consequences of FGM	Good knowledge	1	1
	Poor knowledge	0.58 (0.36-.92)	1.37 (0.63–2.98)
	Have no information	0.79 (0.46–1.36)	1.16 (0.65–2.09)
Knowledge of women on chronic consequences of FGM	Good knowledge	1	1
	Poor knowledge	0.61 (0.38–0.97)	0.98 (0.45–2.12)
	Have no information	0.70 (0.41–1.20)	0.58 (0.56–37)

**Driver, students

*Statistically significant

©Catholics

## Discussion

Despite international and local attempts to end FGM, the practice persists in some parts of the world. FGM was among the traditional practices which are not only prejudicial and harmful to the life of a child but also discriminatory against girls. To get a good understanding of the current practice in the study area, the study assessed both of daughters practice younger than 15 years and women in reproductive age group.

In this study, almost all 729 (97.2%) mothers in the current study expressed that they heard about female circumcision and 159 (21.2%) at 95% (18.3–24.1%) of reproductive age group mothers were circumcised. This finding is far lower than study conducted in Ghana which was 65% [19]. The finding of the current study was also lower than several earlier studies in Ethiopia; Oromia region Bale zone (78.5%) [13], Hararge kersa district (92.3%) [12], Dale Wabera (78%) [20], Somale region Jijjiga district (82.6%) [21], Afar region Gewane Woreda (90.8%) [22], Ahmara region East Gojam zone (62.7%) [23].In similar manner, the this finding of this study was also lower than national prevalence,65% [9].

The discrepancy in findings might be due to study period and socio-cultural status of the study area. Socio-economic status and awareness of the community on harmful traditional practice is increasing over time which might have contributed in lowering the prevalence of FGM. This may also indicate that creating awareness could positively influence ending of FGM practice.

In this study, the prevalence of FGM practice among daughters younger than 15 years was 12 (1.6%) at 95% (0.8–2.5%). The finding of this study is lower than studies done in Oromia region Hararge Kersa district (88.1%), Waliso Woreda (12.8%) [12, 24] and EDHS of 2016, (16%) [9]. The differences in study finding might be due to difference in study period, cultural and religious practice variation of the communities.

The prevalence of FGM among daughters was by far very low when compared with women of reproductive age group. Despite of this much decline magnitude of FGM in the study area, it may still need awareness creation on harmful traditional practice including FGM and law enforcement in the study area to end up FGM. It may also suggest that the due to fear of stigmatization and the legal ban, the finding might have been under reported and authors recommend other exploratory study.

Among the 159 women ever circumcised, 52 (32.7%) reported that they experienced a complication of FGM. Among these, 52 women experienced health consequences of the FGM; excessive bleeding 46 (88.5%), urine retention 30 (57.7%), infection 23 (44.2%), genital swelling 21 (40.4%). This finding was consistent with the study done in Hararge Kersa district which revealed excessive bleeding, urine retention, infection and genital swelling as the common health consequences of FGM [12].

In this study, residence, religion and occupational status of the husband were the identified predictors. Accordingly, Muslim and Protestant follower by their religion less likely practiced female genital mutilation as compared to their counterpart. This finding contradicts with the several studies conducted in Ethiopia: Bale zone [13], Jijjiga district [21] and factor analysis from 2000 and 2005 data [25] which indicated that Muslims were positively significant to FGM practice. The reason for difference might be the distribution of Muslims and Christians is not homogeneous in Ethiopia, and strict traditional and cultural practice of these religion followers is not uniform. FGM might be more common among Christians whereas in another area it might be more common among Muslims, and vice versa. The reason for disparity in finding due to religion also deserves further study.

Women from rural area were seven times more likely practiced FGM as compared to the women from urban residents. This finding was in line with studies conducted in Ethiopia; in Hararge Kersa district, Bale zone, Dale wabera and Jijjiga district [12, 13, 20, 21]. The reason might be due to strong commitment of conserving tradition of socio-FGM practice. The other possible explanation might be due to a relatively loose legal ban of the practice in the rural areas due to sparse population. Furthermore, this might also be explained by of relatively less health information dissemination regarding FGM in rural areas. Authors, therefore suggest that rural area seeks more attention if further reduction of FGM practice is to be practical.

Occupational status of the husband was statistically significant with FGM practice. Women with merchant husbands, daily laborer and others (drivers and students) more likely practiced FGM. This is finding was consistent with the study conducted in Oromia region Dale Wabera district [20]. Individuals with these occupations might have less access to media to get information about FGM practice because of the nature of their occupations. Preparing and distribution of brochures about FGM practice might be helpful for these mobile individuals.

## Conclusion

In this study, the prevalence of FGM practice among daughters and women of reproductive group was by far very low compared to other national study findings. However, the magnitude still seeks attention as Ethiopia has planned to end the practice. Religion, place of residence, and occupational status of the husband were statistically significantly associated with FGM. Strategies to end the FGM practice better should take these variables into consideration.

### Limitation

One of limitation of this study was small sample size. Even though we tried to assure quality of data different mechanisms, fear of stigmatization and legal ban might have played role in under reporting of daughter’s FGM magnitude. Therefore authors invite other exploratory research to counteract incase.

## Data Availability

The data sets and/or analysed during the current study is available from the corresponding author on reasonable request.

## Abbreviations

BSC: Bachelor of Science
CSA: Central Statistical Agency
EDHS: Ethiopian Demographic Health Survey
FGM: Female genital mutilation
SNNPR: South Nations Nationalities, and People’s Region
SPSS: Statistical Package for Social Science
UNICEF: United Nation Children’s Fund
USA: United State of America
WHO: World Health Organization
